# Discovery biology of neuropsychiatric syndromes (DBNS): a center for integrating clinical medicine and basic science

**DOI:** 10.1186/s12888-018-1674-2

**Published:** 2018-04-18

**Authors:** Biju Viswanath, Naren P. Rao, Janardhanan C. Narayanaswamy, Palanimuthu T. Sivakumar, Arun Kandasamy, Muralidharan Kesavan, Urvakhsh Meherwan Mehta, Ganesan Venkatasubramanian, John P. John, Odity Mukherjee, Meera Purushottam, Ramakrishnan Kannan, Bhupesh Mehta, Thennarasu Kandavel, B. Binukumar, Jitender Saini, Deepak Jayarajan, A. Shyamsundar, Sydney Moirangthem, K. G. Vijay Kumar, Jagadisha Thirthalli, Prabha S. Chandra, Bangalore N. Gangadhar, Pratima Murthy, Mitradas M. Panicker, Upinder S. Bhalla, Sumantra Chattarji, Vivek Benegal, Mathew Varghese, Janardhan Y. C. Reddy, Padinjat Raghu, Mahendra Rao, Sanjeev Jain

**Affiliations:** 1 National Institute of Mental Health and Neuro Sciences (NIMHANS), Bangalore, India; 2 Institute for Stem Cell Biology and Regenerative Medicine (InStem), Bangalore, India; 3 National Centre for Biological Sciences, Tata Institute of Fundamental Research (NCBS-TIFR), Bangalore, India

**Keywords:** Endophenotypes, Psychiatry, Pluripotent stem cells, Biorepository, Neuroimaging, Schizophrenia, Bipolar disorder, Obsessive compulsive disorder, Addiction, Dementia

## Abstract

**Background:**

There is emerging evidence that there are shared genetic, environmental and developmental risk factors in psychiatry, that cut across traditional diagnostic boundaries. With this background, the Discovery biology of neuropsychiatric syndromes (DBNS) proposes to recruit patients from five different syndromes (schizophrenia, bipolar disorder, obsessive compulsive disorder, Alzheimer’s dementia and substance use disorders), identify those with multiple affected relatives, and invite these families to participate in this study. The families will be assessed: 1) To compare neuro-endophenotype measures between patients, first degree relatives (FDR) and healthy controls., 2) To identify cellular phenotypes which differentiate the groups., 3) To examine the longitudinal course of neuro-endophenotype measures., 4) To identify measures which correlate with outcome, and 5) To create a unified digital database and biorepository.

**Methods:**

The identification of the index participants will occur at well-established specialty clinics. The selected individuals will have a strong family history (with at least another affected FDR) of mental illness. We will also recruit healthy controls without family history of such illness. All recruited individuals (*N* = 4500) will undergo brief clinical assessments and a blood sample will be drawn for isolation of DNA and peripheral blood mononuclear cells (PBMCs). From among this set, a subset of 1500 individuals (300 families and 300 controls) will be assessed on several additional assessments [detailed clinical assessments, endophenotype measures (neuroimaging- structural and functional, neuropsychology, psychophysics-electroencephalography, functional near infrared spectroscopy, eye movement tracking)], with the intention of conducting repeated measurements every alternate year. PBMCs from this set will be used to generate lymphoblastoid cell lines, and a subset of these would be converted to induced pluripotent stem cell lines and also undergo whole exome sequencing.

**Discussion:**

We hope to identify unique and overlapping brain endophenotypes for major psychiatric syndromes. In a proportion of subjects, we expect these neuro-endophenotypes to progress over time and to predict treatment outcome. Similarly, cellular assays could differentiate cell lines derived from such groups. The repository of biomaterials as well as digital datasets of clinical parameters, will serve as a valuable resource for the broader scientific community who wish to address research questions in the area.

## Background

Severe mental illnesses are a major source of morbidity and disability, with about 2–3% of the population at risk for developing these disorders [1]. These illnesses usually begin in early adult life, with almost 75% of patients developing symptoms by age 24 [2], and often have chronic course. Our current understanding of these diseases suggests that the risk factors that underlie these illnesses have antecedents in early life [3, 4]. In contrast to other chronic medical illnesses, such as cardiovascular diseases or cancers, people with mental disorders become ill at the prime of life and intellectual growth, thus impacting lifespan, quality of life and career [5]. These disorders lead to significant mortality and, morbidity, which requires support at several levels (pharmacological and psycho-social interventions, and rehabilitation). Most interventions are targeted towards amelioration of particular symptoms, as the primary disease processes remain ill understood; and unmet clinical needs remain high [6, 7].

Traditional clinical classification systems conceptualize the psychiatric disorders as a group of discrete syndromes that are independent of each other. For example, schizophrenia, bipolar disorders and other psychotic disorders are considered to differ from each other, based on their unique clinical characteristics, course, family history and treatment response [8]. However, there is now accumulating evidence that there exist overlapping genetic, environmental and developmental factors, cutting across these diagnostic boundaries [9–15]. Cross-disorder consortia studies have attempted to understand the genetic basis of these overlaps and the shared pathophysiology and have found high rates of heritability and co-heritability [16–18]. Many of the genes (and pathways) identified suggest that differences in neural development and connectivity in early life are critical to their pathogenesis. These variations, coupled with epigenetic dysregulation in the brain; influenced by various environmental factors, acting at various points in time during critical neurodevelopmental time windows, influence the onset, and progression, of illness [19]. Disease associated biological signatures are often evident in at-risk individuals of many severe adolescence onset psychiatric disorders (e.g. bipolar disorder (BD) [20, 21], schizophrenia [22], substance use disorders (SUD) [23–25], obsessive compulsive disorder (OCD) [26]) before being manifest as a clinically recognizable syndrome. There is emerging evidence that this also holds true for late onset diseases affecting the brain, and differences in hippocampal structure are evident in childhood or adolescence in persons at risk for Alzheimer’s dementia (AD) [27].

Given these observations, it is necessary that attempts to understand the biology of these disorders considers their genetic basis, differences in neurodevelopment, as well as the overlapping nature of the individual psychiatric syndromes. One way to account for these factors would be to prospectively follow-up affected and unaffected individuals, whose family history suggests an elevated risk of developing disease (based on both clinical information and genetic analysis) from a stage when they are asymptomatic, so that clinical investigations can capture the evolution of alterations in brain function. Such a strategy combined with modern human genetics and analysis of cellular function in brain cells using ‘disease in dish’ models should help us understand the cellular and molecular underpinnings psychiatric illness.

The Discovery biology of neuropsychiatric syndromes [DBNS] is one such research initiative in Bangalore, India, built on a collaboration between clinicians and scientists at the National Institute of Mental Health and Neuro Sciences (NIMHANS), the National Center for Biological Sciences (NCBS), and the Institute for Stem Cell Biology and Regenerative Medicine (InStem). It aims to understand the developmental trajectories and basic biology of these major psychiatric disorders. The study will use multiple techniques (brain imaging, psycho-physics, neuropsychology, next generation sequencing, cellular models), and in-depth clinical assessments of participants in a cohort of multiple affected families with a strong family history of mental illness. The facilities created, and the data sets, will provide a resource for future research to help answer questions with implications for basic neuroscience as well as drive translational research with positive impact for clinical psychiatry.

## Methods

### Rationale

The basic premise of this research initiative is that there exist shared genetic, environmental and developmental factors across the major psychiatric disorders. We aim to identify such overlaps by examining clinical, brain imaging, neurophysiological and neuropsychological measures, as well as clinical histories, across disorders. In addition, we would examine the longitudinal trajectory of these neurobiological measures. We will recruit multiple affected families with patients having one or more of 5 syndromes (schizophrenia, BD, OCD, AD and SUD) so that they broadly represent the major and common psychopathologies. The identification of the index participants will occur at well-established specialty clinics at NIMHANS. Importantly, the index patients chosen will have a strong family history of mental illness. The families will then become part of a single sample, who will be subjected to a uniform set of clinical analyses; and a bio-repository will be set up using cellular material from these individuals. Several endophenotype assessments (neuro-imaging, psycho-physiology, neuropsychology), will be also be performed, as these empirical measures will provide robust and consistent measures of brain function. Familial stress, and psycho-social factors will also be evaluated using structured assessments. It is expected that such deep interrogation of brain networks and sub-cellular networks using molecular and cellular analysis; as well as social and psychological measurements, would lead to better mechanistic understanding of these disorders.

### Objectives

Specific objectives of the program are as follows:

Short-term objectives

- To identify 300 families in whom multiple members (more than 2 affected first degree relatives in a nuclear family) are diagnosed to have a major psychiatric disorder (schizophrenia, BD, OCD, AD and/ or SUD) with structured assessments.
- To study brain structural [grey (magnetic resonance imaging-MRI) and white matter (diffusion tensor MRI)] abnormalities, resting and task-related functional MRI activity, neuropsychological performance, brain electrical activity and eye movement abnormalities in probands with major psychiatric disorders and their unaffected first-degree relatives (FDR); in comparison with matched healthy controls
- To identify cellular phenotypes that differentiate between the groups using experiments on cell lines [lymphoblastoid cell lines (LCL), induced pluripotent stem cell (IPS) lines, IPS derived neurons/glia]
- Create a unified digital database of all the above information.

Long-term objectives (5 years and beyond)

e. To examine the time course and progression of structural/functional brain abnormalities, neuropsychological performance, brain electrical functions and eye movement abnormalities in probands and unaffected FDRs and study their relationship to course of illness and disease conversion.
f. To examine cell lines from good and poor outcome patients to try and identify cellular mechanisms that might underpin or correlate with specific clinical outcomes.

### Hypothesis

In comparison with healthy controls, patients could have significant specific grey matter volume deficits and white matter hypo-connectivity; aberrant neuro-hemodynamic response involving frontal, striatal & limbic brain regions during fMRI; abnormal brain electrical activity and antisaccade/ smooth pursuit eye movements; prominent and specific patterns of impairments in verbal memory, verbal fluency, sustained attention and executive functions.

Unaffected relatives could also have brain abnormalities; and a proportion of these individuals may develop a clinical syndrome. A composite endophenotype comprising of neuroimaging and neurocognitive parameters could differentiate three groups (affected subjects, unaffected relatives & matched healthy controls). Subjects could show the deficits even in periods of clinical remission; and in a proportion of severely ill subjects these deficits may progress over a period.

Cellular assays could similarly differentiate cell lines derived from the three groups. Greater brain abnormalities/composite endophenotype measures/cellular abnormalities at baseline may predict poorer course and outcome/ treatment response. The relation between these, and the genetic variations, would be amenable for further analysis, to understand the genotype-phenotype conversion.

### Clinical recruitment and work-plan

The affected probands will be recruited from the adult psychiatry services and specialty clinics (center of addiction medicine clinic, schizophrenia clinic, OCD clinic and geriatric clinic) of the NIMHANS, Bangalore. Unaffected FDRs will also be recruited from the families. An attempt will be made to recruit as many individuals as possible from each family. All clinics will together recruit age and gender matched control subjects, who will neither have an axis I psychiatric disorder, nor have a family history of such disorders in two previous generations.

All recruited individuals (*N* = 4500) will undergo brief clinical assessments and a blood sample will be drawn for isolation of DNA and peripheral blood mononuclear cells (PBMCs). From among this set, a subset of 1500 individuals (300 families and 300 controls) will be assessed at baseline on several additional assessments [detailed clinical assessments, endophenotype measures (neuroimaging, neuropsychology, psychophysics)], with the intention of conducting repeated measurements every alternate year. These individuals will form the neurodevelopmental endophenotype cohort (NEC). PBMCs from the NEC will be used to generate lymphoblastoid cell lines and a subset of this would be converted to IPS lines and also undego whole exome sequencing.

### Specific methods

#### Clinical assessments

The study includes two levels of assessments - brief assessments and the NEC assessments. Brief, standardized assessments of overall health with a view to recording any pre-existing medical conditions or co-morbidity will be performed on all individuals who consent to participate in the study. This will provide diagnostics and psychometric evaluations that are compatible with international practice, and allow comparisons to be drawn. In addition, a more extensive endophenotype assessment will be performed on those families consenting to be part of the NEC. For the eligible subjects who consent for the endophenotype assessments, further detailed clinical evaluation of brain function will be done. This will include cross-disorder measures to assess temperament, personality, adverse childhood experiences, life events, handedness, socioeconomic status, functioning and psychopathology-specific scales. All clinical assessments that will form part of the assessment of the NEC are listed in Table 1.

**Table 1:** Systematic data recorded during recruitment and two-yearly follow-ups in ADBS

Sociodemographic data	Age, gender, education, language, ethnicity/region of origin
Brief assessments	Psychiatry clinical work-up sheet with history of the presenting illness, detailed assessment of the family pedigree, developmental history assessment and mental status examination.
	Medical history
	History of previous medications – effects and adverse events
	Physical examination
	DSM V cross-cutting diagnostic assessment [73]
	M.I.N.I. international neuropsychiatric interview (Version 5.0.0) [74]
	Family interview for genetic studies (FIGS) [75]
	Adult ADHD self-report scale (version 1.1) [76]
	Hindi mental state examination (HMSE) [77]
	Clinical global impression severity rating (CGI-S) [78]
Neurodevelopmental endophenotype assessments – clinical (general)	Adult temperament questionnaire [79]
	20 Item mini-IPIP [80]
	Global assessment of functioning (GAF) [81]
	Work and social adjustment scale (WSAS) [82]
	Adverse childhood experiences international questionnaire (ACE-IQ) [83]
	Interview for recent life events (IRLE) [84]
	Kuppuswamy scale [85]
	Edinburg handedness inventory [86]
Neurodevelopmental endophenotype baseline assessments – clinical (psychopathology specific)	Schedule for assessment of positive symptoms (SAPS) [87]
	Schedule for assessment of negative symptoms (SANS) [88]
	Young’s mania rating scale (YMRS) [89]
	Hamilton depression rating scale (HDRS) [90]
	Hamilton anxiety rating scale (HARS) [91]
	Yale-Brown obsessive compulsive scale [92]
	Clinical dementia rating Scale [93]
Neurodevelopmental endophenotype assessments – cognitive	Digit Forward Span (attention and concentration) [94]
	Color trails 1 (processing speed) [95]
	Verbal N Back 1&2 (working memory) [96]
	Color trails 2 (cognitive flexibility) [95]
	Stop signal task (response inhibition) [97]
	Rey-Osterrieth Auditory Verbal Learning Test (verbal learning and memory) [98]
	2nd order theory of mind stories (theory of mind) [99]
Neurodevelopmental endophenotype assessments – neuroimaging	T1-weighted structural magnetic resonance imaging (MRI)
	Structural White Matter- Diffusion Tensor Imaging (DTI) using an echo planar imaging sequence
	Rest-functional MRI
	Task-functional MRI: emotion processing [31, 100]
	Task-functional MRI: word generation [33]
Neurodevelopmental endophenotype assessments – psychophysics & clinical electrophysiology	Eye-tracking studies – saccade experiments [101] and smooth pursuit eye movement (SPEM) experiments [102]
	Functional Near Infrared Spectroscopy (fNIRS): resting state and during cognitive experiments (tower of London task [103] & facial emotion processing task)
	Electroencephalography – resting state [35] and P50-suppression [104]

#### Endophenotype assessments

The development of obvious clinical features in psychiatric diseases is usually preceded by a long period during which there are likely to be changes in brain function that contribute to disease development. We propose to map the development of these changes in the NEC using a number of complementary approaches. Previous research from NIMHANS and elsewhere have identified structural & functional brain imaging, eye movement parameters, electroencephalography (EEG) measures and cortical hemodynamic changes as robust endophenotypes [23, 26, 28–42] However, these assessments have largely been performed as single time measurements in patients with fully developed disease, their relatives and controls. These studies demonstrate that patients and their relatives differ from control subjects on these parameters. To capture the evolution of these important quantitative endophenotypes during critical stages of disease development, from the asymptomatic state, through to various stages of clinical disease, we propose to perform these measurements at two-year intervals in patients, unaffected FDR and healthy controls. The NEC assessments are outlined in Table 1.

#### Next-generation sequencing

**Fig. 1:**
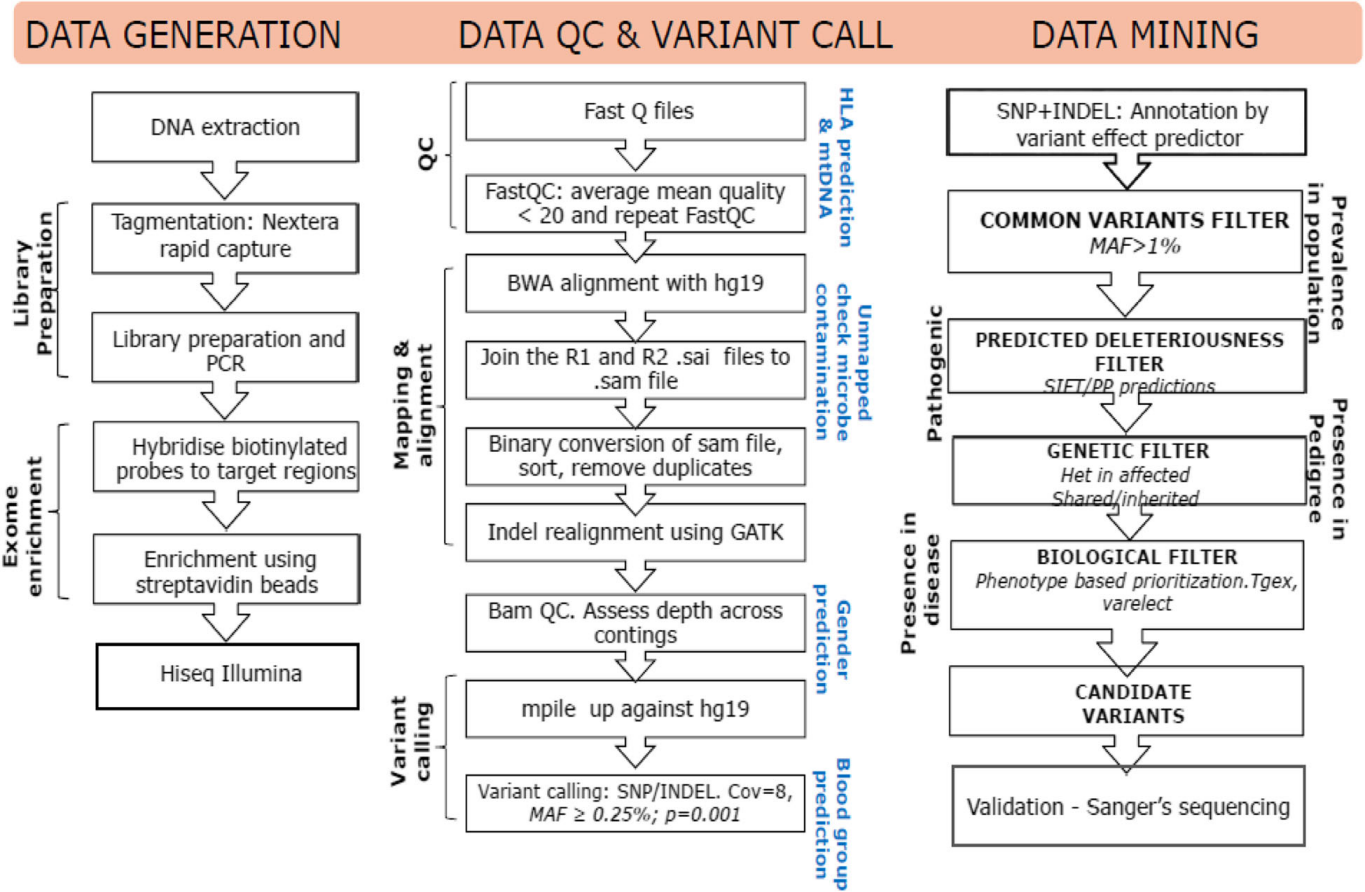
Whole exome sequencing

Heritability estimates for severe mental illnesses range from 60% - 90%, suggesting that a large proportion of etiological variance is attributable to genetic factors [43–45]. Despite this evidence, additive effects of loci implicated by large scale genome-wide association studies (GWAS) have been estimated to explain only a small portion of this liability [46]. This has resulted in a shift in focus from common variants of small effect to rare and de-novo variants as determinants of disease causation. The rapid evolution of next generation sequencing platforms and falling costs of sequencing have enabled the identification of some of these rare and de-novo variants. Families with multiple affected members across generations are a critical resource as they have greater probability of segregating these putative causative variants for severe mental illness. The standard operating procedure (SOP) for next-generation sequencing in DBNS is given in Fig. 1. During this study, we propose to perform whole exome sequencing from affected individuals and their unaffected family members. The availability of such sequence along with suitable bioinformatics analysis will facilitate the identification of novel variants that may segregate with disease or specific aspects of illness such as responsiveness or resistance to specific treatment.

#### Cellular models: National Biorepository for mental illness

**Fig. 2:**
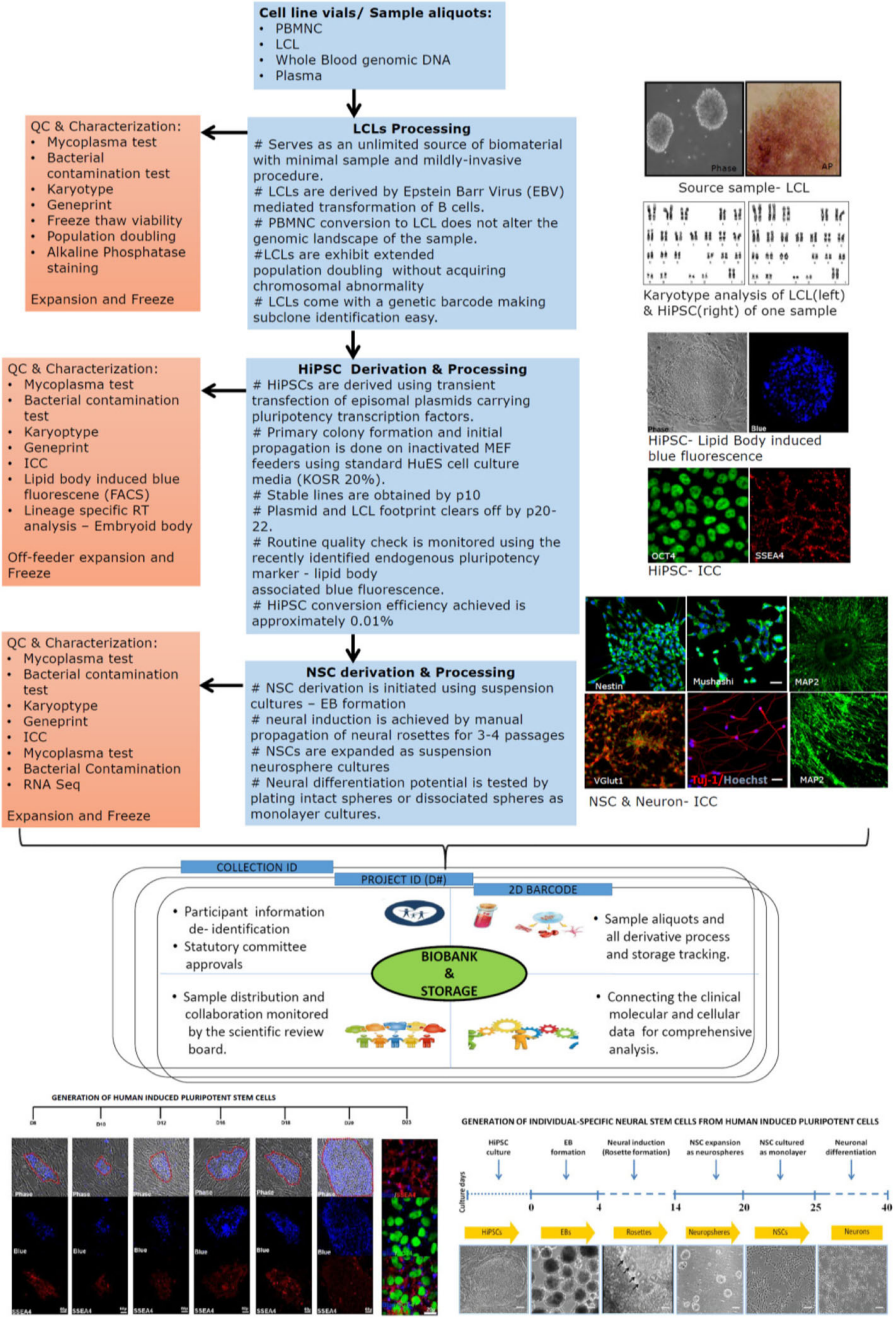
Biomaterial processing: Peripheral blood mononuclear cells (PBMNC) are extracted from blood. These cells are converted to lymphoblastoid cell lines (LCLs), which are then subsequently reprogrammed to generate human induced pluripotent stem cells (HiPSC). The HiPSCs are further transformed to neural stem cells (NSC). Quality control (QC) and characterization at each step is shown [49]

Critical to understanding the evolution of neuropsychiatric syndromes is the unravelling of cellular and sub-cellular processes that are altered in a given syndrome. This poses a unique challenge in the study of the brain related diseases, as the tissue is inaccessible. The availability of living brain cells is critical to study ongoing cellular processes in neurons and glial cells. Recent developments in stem cell technology allow the use of somatic tissues to be reprogrammed into IPS lines. These can then be differentiated into neural cells including neurons and glia. We propose to use this approach to derived cellular models of disease in the context of neuropsychiatric illness. Methods have been developed, that allow the establishment of stable cell lines from lymphocytes isolated from a sample of peripheral blood [47]. These LCLs can then be converted to IPS lines using non-integrating plasmids, which are free of transgene footprints and ideal for modelling disease mechanism [48, 49]. IPS lines can be differentiated into specific neuronal/glial cell types as well as composite cultures using a variety of available methods. Study of cellular and molecular processes in patient derived neuronal cell lines of a defined genetic background can be correlated with documented clinical semiology and the detailed endophenotype data. The SOPs for biomaterial processing is given in Fig. 2.

The functional unit of the brain is the synapse and it is likely that alterations in synapse formation, structure and function underlie neuropsychiatric illness. Synapse development and function is underpinned by complex sub-cellular processes and the “disease in a dish” model offers a unique opportunity to understand these events and their role in mental illness. To functionally characterize the IPS derived neural cell models we will test for neuronal activity. This will be done using whole cell patch-clamp recordings to test for the ability of neurons to generate action potentials. In addition, we will also measure function through the measurement and analysis of calcium transients using fluorescent calcium indicator dyes. More detailed analysis of response to neurotransmitters can also be performed using these approaches. In addition, neural networks will be examined in cultures using multi-electrode arrays.

Given the neurodevelopmental nature of mental illness, it is likely that altered synapse formation and pruning plays a role in disease development. To understand this process, we propose to characterize synapse development in our cellular models. This can be done by differentiating IPS into neurons in cell culture and characterizing neurite outgrowth during this process using well-established quantitative assays.

Metabolic alterations in neural cells are strongly implicated in mental illness and multiple roles for mitochondrial dysfunction in the development of several mental disorders is widely discussed. To map these potential defects, we will perform basic characterization of mitochondrial structure and function using well-established fluorescence based assays. Metabolic alterations arising from altered mitochondrial function or additional genetic factors will be captured by metabolic profiling using small molecule mass-spectrometry. Such analyses may also include changes in the levels of neurotransmitters.

#### Sample size calculation and statistical analysis

The sample size was calculated for each trait and number of times of assessment separately for each trait [50] for repeated measurements. The required number of families (n) assuming independence of subjects, can be obtained using [50], as follows.

Let σ^2^ is the assumed common variance in the two groups, (μ_1_ − μ_2_) is the difference in means of the two groups, k is the number of time points and ρ is the assumed correlation of the repeated measures; then the number of subjects/ families (assuming independence) in each of two groups is,

$$ \mathrm{n}=\frac{2{\left({\mathrm{Z}}_{1-\frac{\upalpha}{2}}+{\mathrm{Z}}_{1-\upbeta \kern0.75em }\right)}^2\ \left[1+\left(\mathrm{k}-1\right)\uprho \right]}{\mathrm{k}\ {\left[\left({\upmu}_1-{\upmu}_2\right)/\upsigma \right]}^2\ } $$

The power and type I error were kept at 80% and 5% respectively.

If the affected group and high-risk group (FDRs) have an average family size of ‘m’, thereby the total number of observations = ‘nm’, then the effective sample would be N = nm/[1 + (m-1) λ]; where ‘λ’ is the intra-familial correlation (intra-class correlation) on the quantitative trait.

We have used this N in the standard power computation formula for unbalanced ANOVA for the number of observations in the affected group and FDRs along with the control group.

Using this formula, we have calculated sample size using brain imaging and brain physiology measures. These are as follows:

- Corpus callosal volume: This white matter tract has been implicated in all major psychiatric disorders and has also been examined in large scale studies. Using published literature on corpus callosal volume [51], sample size calculation as per above formula reveals 1509 participants overall for 80% power.
- Eye movement parameters: Eye movement abnormalities have been identified as putative endophenotypes in recent large studies [52]. Sample size calculated for antisaccade latency was 1257 participants overall for 80% power.

These power calculations described above will adequately address our capacity to detect group differences. Moreover, as we detect genetic differences (rare variants/ accumulation of damaging variants), and also submit the genetic data generated to pathway based modelling, these emerging data will be used to analyse MRI and EEG data in a specific manner.

## Discussion

### Longitudinal assessment of biological measures

The human brain is a complex organ and its output, i.e. manifest behavior, arises from processes operating at multiple scales ranging from individual molecules within cells and integrating into increasingly higher levels of complexity including cell- cell interactions and circuit formation leading to emerging structure and function. These multi-scale events are underpinned not only by genetic factors that determine the functional biochemistry of brain cells, but also by epigenetic factors that modulate the expression of genes in an individual genome. Given the inherent variability between the genome of individuals as well as the range of environmental factors that can influence gene expression, it is not surprising that uncovering the etiology and pathogenesis of mental illness remains one of the greatest challenges in modern biology. To meet this challenge, our proposal to assemble a cohort of clinically dense families and prospectively follow the development of disease in them, would be useful. The use of clinically dense families coupled with next generation sequencing offers an opportunity to uncover rare and de novo variants and study their segregation pattern within a family. These observations could offer insights into the inherited basis of mental illness, as also be extrapolated to the whole population. Simultaneously, the detailed clinical history and demographics collected for members of the cohort will offer an insight into environmental factors that may mediate epigenetic regulation of the genome in an individual and therefore explain the development of mental illness [53].

Numerous case-control studies have identified changes in brain structure and function at multiple levels. However, we are unaware of any study which has mapped the development of such changes from at-risk state to disease onset, using multiple measures. Most of our knowledge of brain perturbations that lead to psychopathology has been derived from cross-sectional “snap-shots”, or explorations which commence after the development of a recognizable syndromal state. The specific roles that genes, environment and their interaction play in typical and atypical development are unclear, especially across the course of mental illness. Existing knowledge of brain and behavioral development is insufficient to permit identification of specific time points at which normal and psychopathological trajectories diverge and the processes that underlie such deviations. We propose to set up a prospective longitudinal cohort of individuals who are at-risk of developing psychiatric disorders and follow them with periodic endophenotype assessment over time. This approach offers the opportunity to map structural and functional changes in brain function as they develop, along with behavioral changes that lead to disease development. This is likely to be vastly more informative than previous cross-sectional case-control studies. We will use advanced imaging methods to accurately track, changes in brain structure and connectivity (both structural and functional), the neurodevelopmental processes related to the normal developmental sequence and deviations from it. In contrast to conventional genetic association studies where disease status is based on clinical observations, neuroimaging phenotypes can capture aspects of disease phenotypes at the physiological level. Neuroimaging genetic studies thus offer the prospect of gains in statistical power, since genes code not for mental or behavioural traits, but for the neural phenotypes that underpin them [54]. Neuroimaging genetic studies have the additional benefit of spatially localizing gene effects, offering further potential insights into the structural and functional neurobiology of disease [55–58]. Thus, the availability of this temporal data set may offer predictive value in relating specific changes in brain structure and function to the development of clinical features or response to treatment. Uncovering such changes may offer clinical psychiatry the opportunity to personalize treatment; e.g. when a specific early change in brain structure or function predicts a clinical outcome. They may also offer the opportunity to predict treatment response and therefore offer personalized medicine.

### Aggregation of syndromes with varied symptomatology

The validity of the discrete disease constructs (constructed mainly based on phenomenological similarities and dissimilarities) and classificatory models of mental disorders, which have hitherto been adequate for clinical purposes, have been strongly challenged by recent findings of wide overlaps between these discrete entities in terms of genetic underpinnings, neurodevelopmental substrates, responses to pharmacological agents and behavioural treatments [9–15]. In fact, considerable overlap is present in the phenomenology as well [9, 59]. Moreover, these disorders do not necessarily breed true and it is not uncommon to find that an index case may have a family history of a different psychiatric illness [59–61]. This lack of validity of a symptom based classification is considered as one of the main reasons for the absence of diagnostic specificity of biological observations [62]. It is likely that the recognizable, syndromal disease states are due to deviations on the central trajectory of the normal brain developmental sequence which includes processes like neuronal migration, proliferation, pruning and myelination.

By recruiting individuals from different disorders and mapping endophenotypes across different domains, we hope that we will be able to achieve increased power to identify the common neurobiological factors underlying severe mental illness. Such attempts have been made by recent research consortia like the Bipolar-Schizophrenia Network for Intermediate Phenotypes (B-SNIP) and Consortium on the Genetics of Schizophrenia (COGS) [10, 11, 63]. While these initiatives were restricted to focused on the psychosis spectrum, in the present study we aim to investigate beyond psychosis spectrum; and additionally, use cellular models.

Of the neuropsychiatric disorders that we have included in this study, the outlier is dementia, which usually manifests at a later age than the others. However, dementia is also increasingly recognized to have its beginnings in adolescence, as early studies point to recognizable brain changes in persons at high risk, starting many years before the recognizable symptoms of the disease are palpable [64–67]. Moreover, overlaps in symptoms, risk to family members and genetic factors are evident [68, 69]. Such findings support the inclusion of AD patients in a cohort of neurodevelopmental disorders.

### Combination of clinical endophenotypes and IPS

It is now possible to assemble and manipulate so-called neural spheroids, generated from human induced pluripotent stem cells, to study the normal development of the human brain and deviations from the developmental sequence. Since many neuropsychiatric disorders are possibly influenced by individual genetic influences, it is difficult to study these diseases in standard animal models. Instead, these types of diseases can be modeled using the cells from the patient. One can use the information from longitudinal tracking of the basic processes underlying brains fundamental neurodevelopmental sequence in normal development and in disease, to a] investigate the internal cellular processes, underlying the processes involved in neurodevelopment, b] Model, deviations in cellular processes, which lead to deviations in neurodevelopmental processes, and c] test, the processes and outcomes of physical and drug treatments.

### Resources to be generated and expected outcomes

The unified digital database of clinical-endophenotype measures and the bio-repository generated in DBNS will provide a wealth of quantitative detail, which can then be interrogated both within and across phenotype (clinical overlaps/outcome) as well as genetic data (presence of at-risk alleles, or sharing). Other than the specified objectives, some of the other expected off-shoots of the project are as follows:

#### Genetic basis of psychiatric endophenotypes

The program seeks to identify valid endophenotypes in psychiatry using a large sample; and have next generation sequencing data for the same set of individuals. Using appropriate statistical techniques for data reduction (e.g: principal components analysis) and endophenotype-genetics relationships (e.g: parallel independent component analysis), we hope to identify the genetic basis of these endophenotypes. Similar endophenotype-genetics analyses (*n* = 1250) have been recently published from the B-SNIP cohort [37, 70].

#### Mechanisms/predictors of drug response/adverse events

Availability of clinical-endophenotype measures and biomaterial from individuals who have/haven’t responded to particular drugs/ have adverse events to specific drugs will be valuable tools in this approach. Cellular models from such individuals can also be used for testing drug effects in-vitro and for the identification of new drug targets [71]. Such an approach has already been used for Lithium response in bipolar disorder [72] and needs to be extended to other disorders and drugs.

#### Gene editing experiments

We hope to identify damaging rare variants in the loaded families using next generation sequencing. Advances in genome engineering techniques based on the CRISPR-associated RNA-guided endonuclease Cas9 allow us to create isogenic cell lines, either by inserting these mutations into a control cell line, or by correction of the mutations in a cell line from a diseased individual. The advantage of such an approach is that each modified cell line will have its own control, the only difference being the edited mutation with the genomic background unaltered.

#### Combination of cellular models

Many of the biological signals being identified in psychiatry indicate involvement of systemic genes. The use of IPS derived neuronal lineage cells alone may not capture these systemic effects; hence, a combination of IPS derived neurons, glia, and peripheral model systems (e.g., LCLs, lymphocytes) will prove useful. Additional use of peripheral cells will also advance bench to bedside translation.

#### Biological predictors of disease conversion

The unique feature of this program is the combination of a clinical longitudinal approach with prospective assessment of biological measures. A proportion of the FDRs will convert to clinical illness during the follow-up period. The program should be able to identify measurable predictors of disease conversion in FDRs at various biological levels – brain imaging, psycho-physics, epigenetics etc. In addition, examination of cellular endophenotypes across FDRs who have converted to clinical disease and who have not, in the same family, may point towards disease conversion related functional output at the cellular level.

## Conclusion

The DBNS is thus an ambitious, and optimistic, attempt to create a database that combines a wealth of clinical data with a comprehensive psychological and biological assessment, over time. The resources generated will hopefully serve as a platform to answer several questions related to the neurobiology of psychiatric disorders; as well as address fundamental questions about neuro-development and degeneration, as relevant to common psychiatric disease syndromes. This would improve our understanding of these illnesses, and perhaps develop strategies for amelioration, of these conditions which are a significant public health concern.

## Abbreviations

AD: Alzheimer’s dementia
ADBS: Accelerator program for discovery in brain disorders using stem cells
BD: Bipolar disorder
DBNS: Discovery biology of neuropsychiatric syndromes
EEG: Electroencephalography
FDR: First degree relative
GWAS: Genome-wide association study
IPS: Induced pluripotent stem cell
LCL: Lymphoblastoid cell lines
MRI: Magnetic resonance imaging
NEC: Neurodevelopmental endophenotype cohort
OCD: Obsessive compulsive disorder
PBMC: Peripheral blood mononuclear cell
SOP: Standard operating procedure
SUD: Substance use disorder
